# Examining the Relationship Between Alopecia Areata and Mental Health: An Investigation of the Global Burden of Disease Study 2021

**DOI:** 10.1111/jocd.70325

**Published:** 2025-06-30

**Authors:** Aditya K. Gupta, Vasiliki Economopoulos, Mesbah Talukder

**Affiliations:** ^1^ Division of Dermatology, Department of Medicine Temerty Faculty of Medicine, University of Toronto Toronto Ontario Canada; ^2^ Mediprobe Research Inc. London Ontario Canada; ^3^ School of Pharmacy, BRAC University Dhaka Bangladesh

**Keywords:** alopecia areata, global burden of disease study 2021, mental health

## Abstract

**Background:**

Alopecia areata (AA), an autoimmune hair loss disorder, can significantly alter a person's appearance and cause emotional distress. This disorder has been linked to anxiety and depression, but most work has been done on either one‐population samples or has been conducted using heterogeneous populations, potentially skewing results.

**Aims:**

We aim to obtain a better understanding of the relationship of AA with anxiety and depression in more finely divided populations based on sex, age, and country.

**Methods:**

We have accessed data present on AA, anxiety and depressive disorders within the Global Burden of Disease Study 2021. We downloaded data from China, Japan, India, Brazil and the United States for males and females less than 20, 20 to 54 and 55 years of age and older. We extracted the prevalence and years lived with disability (YLDs) measures as surrogate markers for extent and severity of disease respectively. Pearson's correlation coefficient was calculated for both prevalence and YLDs for AA versus anxiety as well as for AA versus depression.

**Results:**

We found significant positive correlations of AA with anxiety and depression in females: primarily in China, Japan, India, and Brazil for anxiety, and China, India, and Brazil for depression. Additionally, significant correlations tended to occur in younger females.

**Conclusions:**

This study demonstrates differences in the correlation of AA disease extent and severity with anxiety and depression between countries, sex, and age. This highlights the need for more finely detailed studies to truly determine the impact of AA on mental health globally.

## 1 Introduction

Hair loss due to alopecia areata can be an extremely anxiety‐provoking experience for those affected by the condition and their families [1]. This disorder affects approximately 2% of the global population, with women typically twice as likely to have alopecia areata [2]. This hair loss disorder is known to be associated with other comorbid conditions, and while many studies have investigated the rates of comorbid disorders in alopecia areata, few have specifically examined the relationship between mental health disorders and alopecia areata.

A survey study of over 19,000 individuals across 27 European countries on the impact of skin disorders on quality of life demonstrated that those with any type of alopecia reported substantial levels of anxiety or depression [3], while a separate European study examining alopecia areata and androgenetic alopecia independently found that both of these disorders are associated with reduced quality of life, specifically in anxiety and depression measures [4]. A study of the All of Us research program in the United States demonstrated that patients with alopecia areata are more likely to have alcohol use disorder, attention deficit hyperactivity disorder, and insomnia [5], while another study of the same dataset demonstrated higher rates of both anxiety and depression in alopecia areata patients [6]. Additionally, studies have shown that there is a stigma surrounding hair loss, which can be influenced by the portrayal of patients with hair loss in media [7, 8, 9].

Of those studies that have examined these relationships, they have involved either one population from a single country [5] or examination of the relationship on a global scale, including many socio‐culturally diverse populations [2, 10]. The drawback of the one‐population approach is that the overall trends cannot be generalized to other populations, and the main drawback of the second global approach is that by combining data from multiple countries/regions/populations—such as those performed by Gisondi et al. and Titeca et al. [3, 4]—the results that are obtained may not be applicable to each individual country/region/population that was included in the study, as cultural variations, particularly surrounding stigma and cultural attitudes towards hair loss, would not be accounted for.

The main goal of this study was to determine the relationship between alopecia areata and anxiety and depressive disorders in different countries with varying social norms by both age and sex.

## 2 Materials and Methods

All data examined in this study came from the Global Burden of Disease Study 2021, which contains aggregate population data for a wide variety of diseases and disorders from countries across the globe [11]. No institutional approval was required for this current study, as we are working with aggregate data available to the general public accessible at https://vizhub.healthdata.org/gbd‐results/.

We obtained aggregate data on the prevalence (rate per 100 000 individuals) and years lived with disability (YLDs, per 100 000 individuals) measures for alopecia areata, anxiety disorders and depressive disorders for males and females under the age of 20 years, between the ages of 20–54 years and over the age of 50 years. We included data from China, Japan, India, Brazil and the United States.

All data was imported into SAS Studio 3.81 (Statistical Analysis Software Institute Inc.; Cary, NC) where the data was analyzed using linear regression, calculating the Pearson's correlation coefficient (*R*), *R* ^2^, and regression fit line. We specifically compared the relation between alopecia areata and anxiety disorders or depressive disorders for both prevalence and YLDs to determine if these conditions increase or decrease in conjunction with each other and how this might vary between countries and regions. From herein we refer to anxiety disorders as anxiety and depressive disorders as depression.

## 3 Results

**FIGURE 1:**
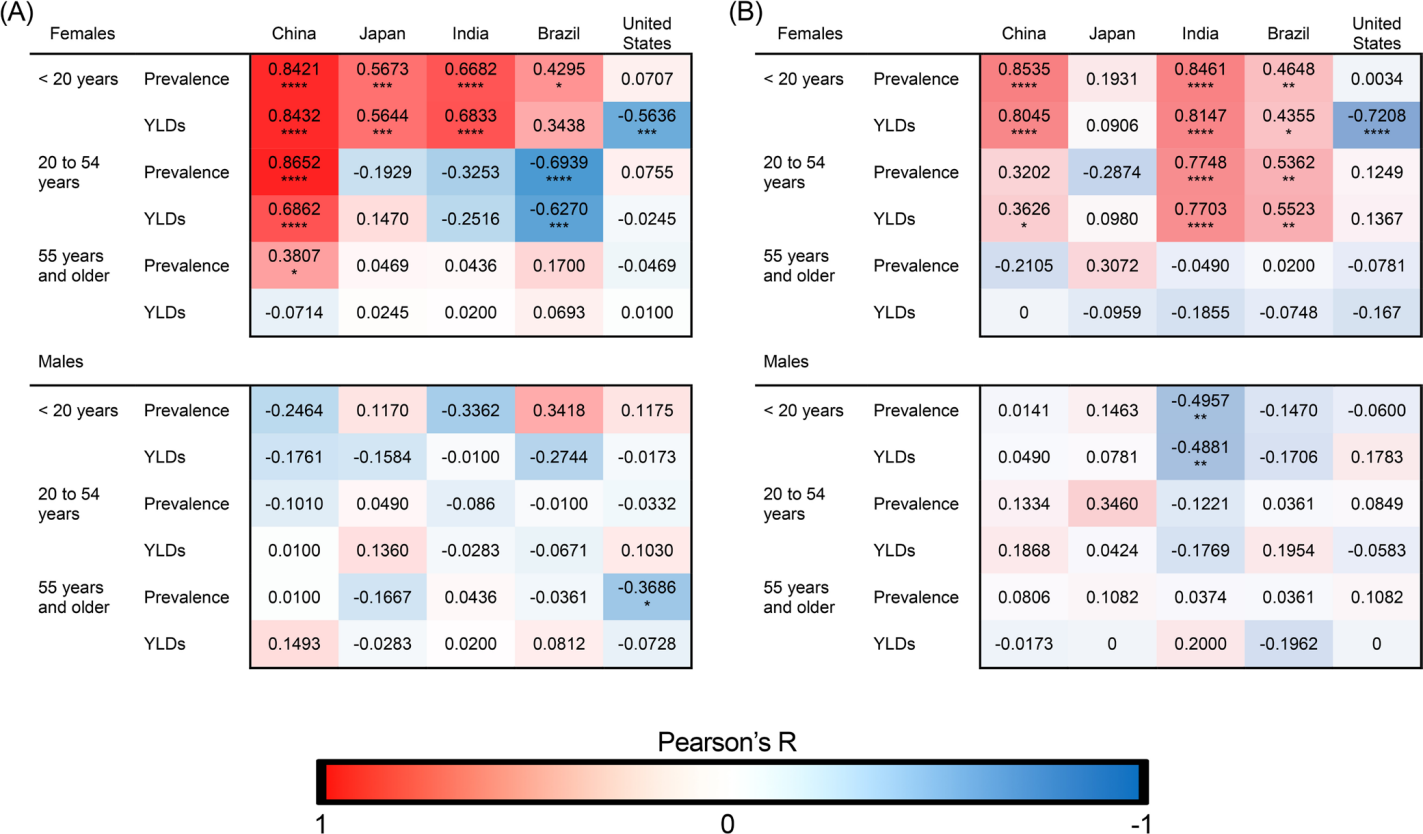
Heat maps of Pearson's correlation coefficient (Pearson's *R*) for alopecia areata and (A) anxiety and (B) depression in all age groups and countries. Red shading represents positive correlations and blue shading represents negative correlations. *p < 0.05; **p < 0.01, ***p < 0.001, ****p < 0.0001.

**FIGURE 2:**
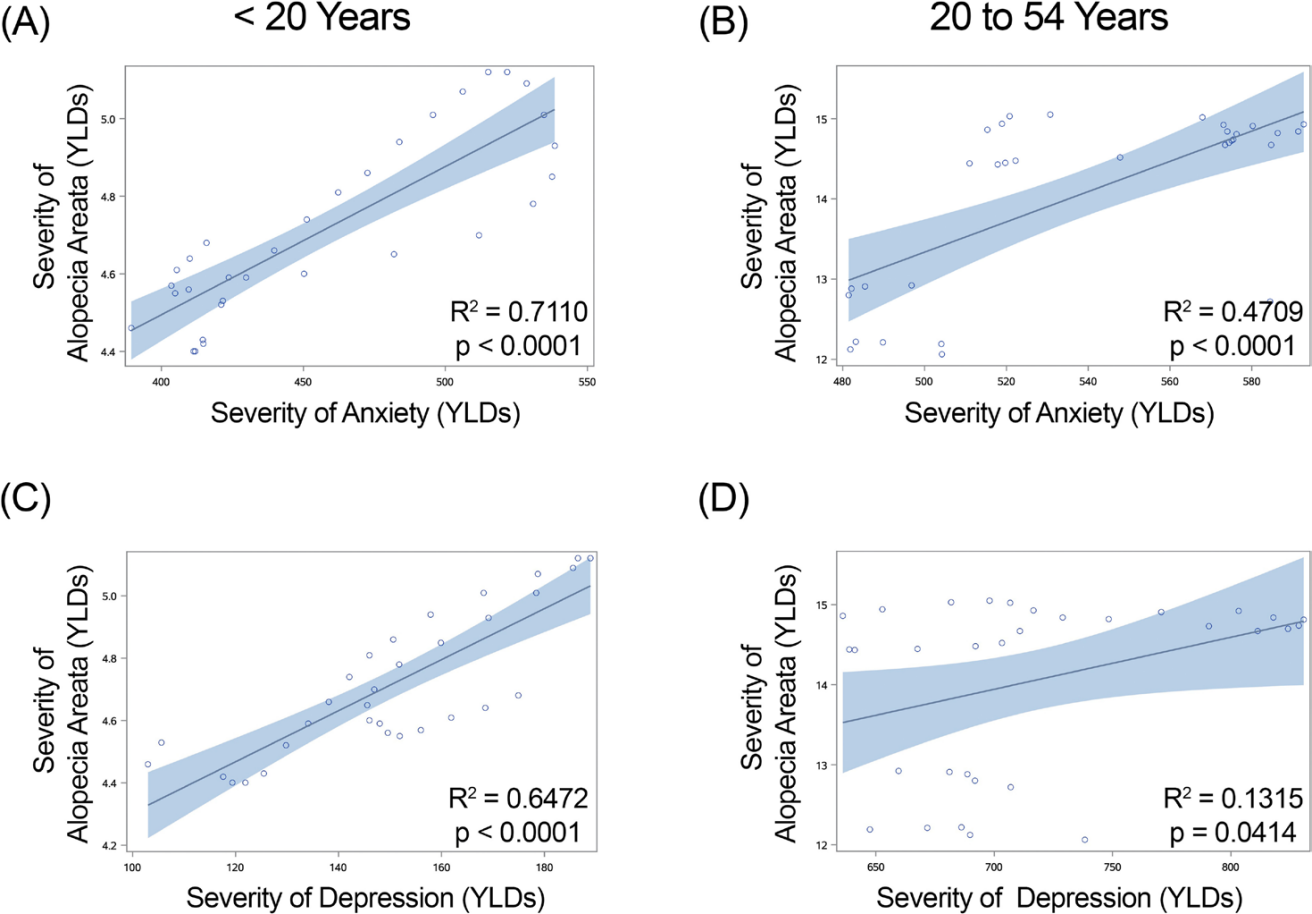
Regression of severity (Years Lived with Disability [YLDs]) of alopecia areata versus anxiety and depression in females within China. Anxiety is shown in graphs (A, B) and depression in graphs (C, D). (A, C) females under 20 years of age; (B, D) females 20–54 years of age. *R* ^2^ and *p*‐values for each regression shown within each graph.

**FIGURE 3:**
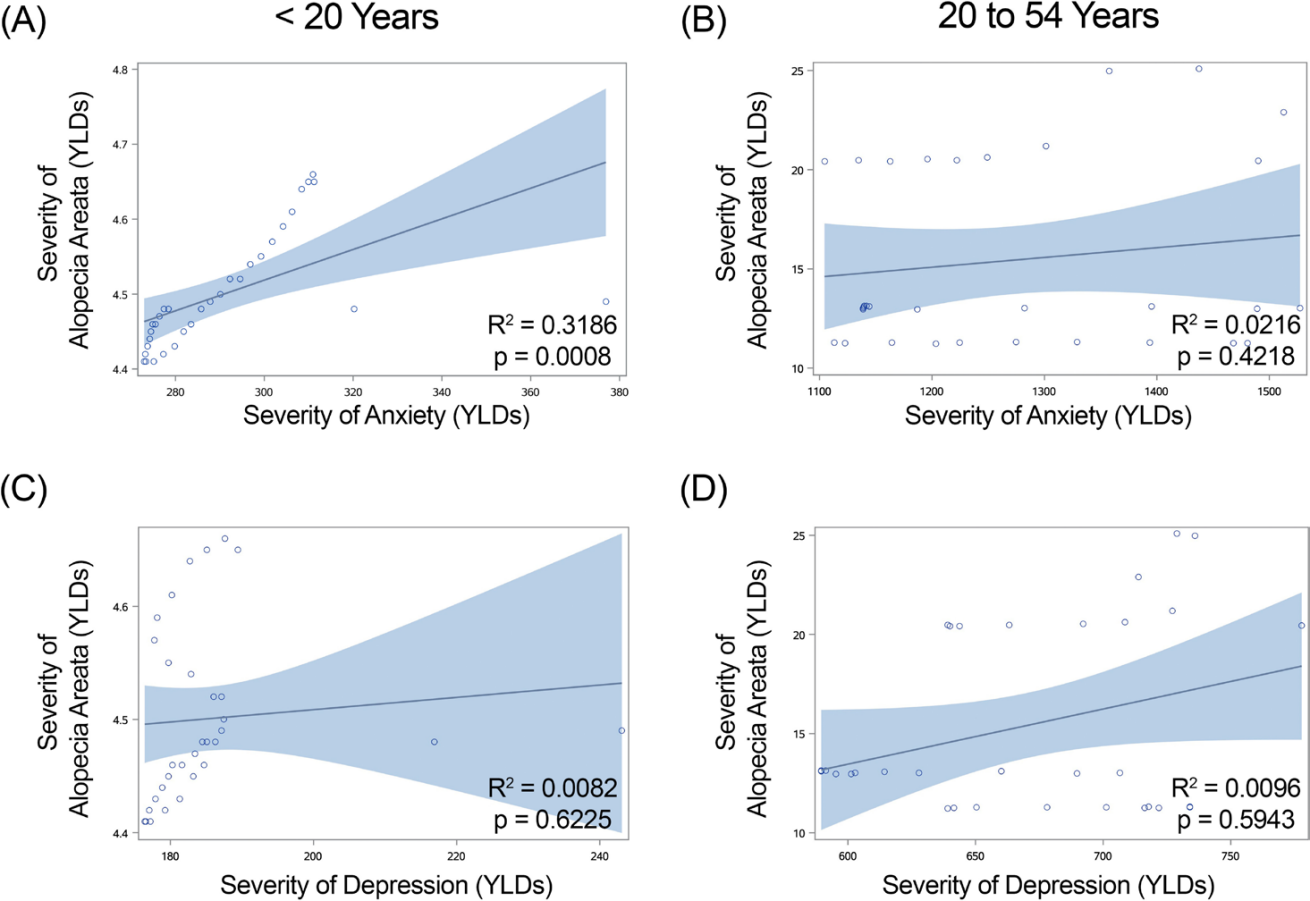
Regression of severity (Years Lived with Disability [YLDs]) of alopecia areata versus anxiety and depression in females within Japan. Anxiety is shown in graphs (A, B) and depression in graphs (C, D). (A, C) females under 20 years of age; (B, D) females 20–54 years of age. *R* ^2^ and *p*‐values for each regression shown within each graph.

**FIGURE 4:**
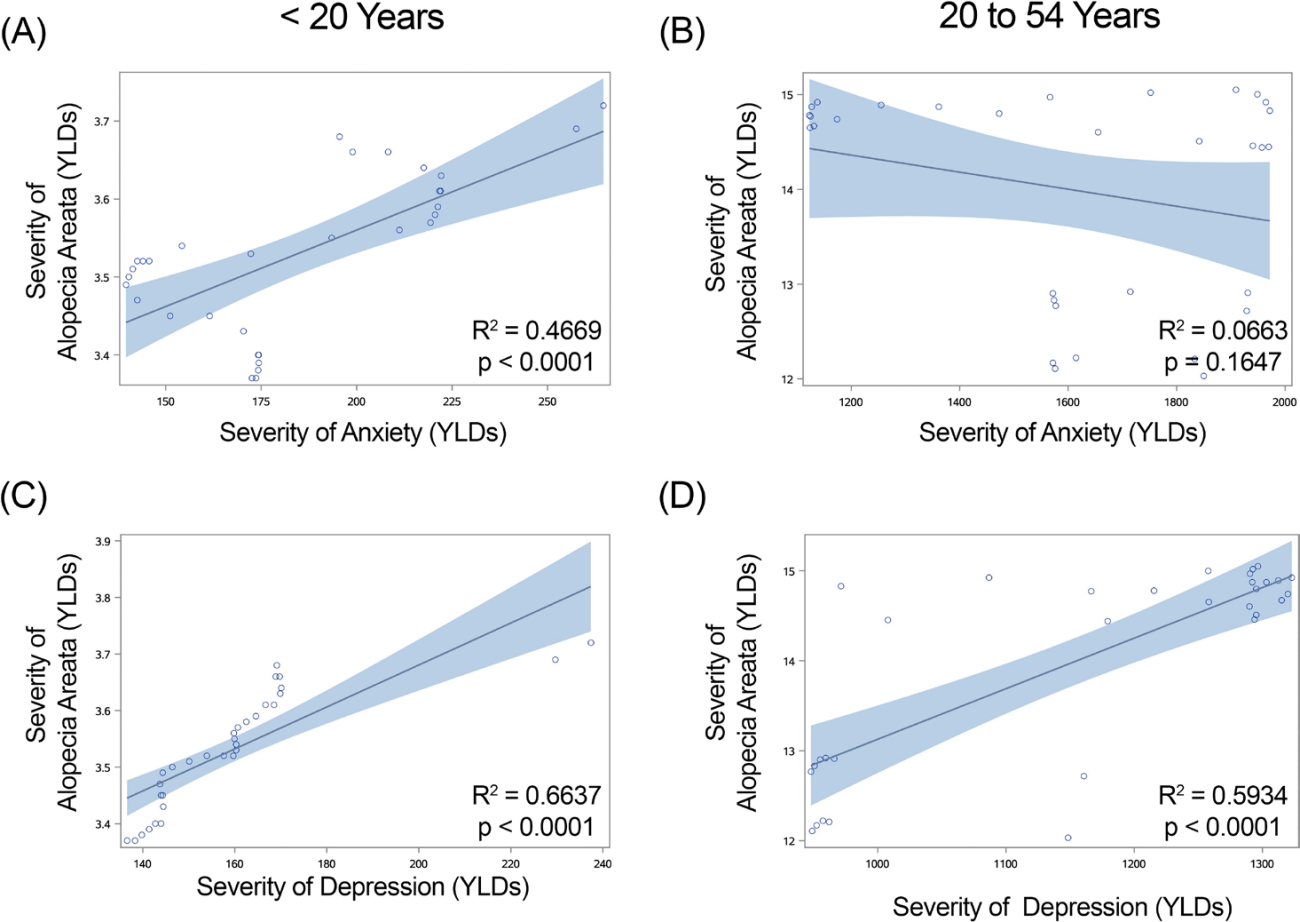
Regression of severity (Years Lived with Disability [YLDs]) of alopecia areata versus anxiety and depression in females within India. Anxiety is shown in graphs (A) and (B) and depression in graphs (C, D). (A, C) females under 20 years of age; (B, D) females 20–54 years of age. *R* ^2^ and *p*‐values for each regression shown within each graph.

**FIGURE 5:**
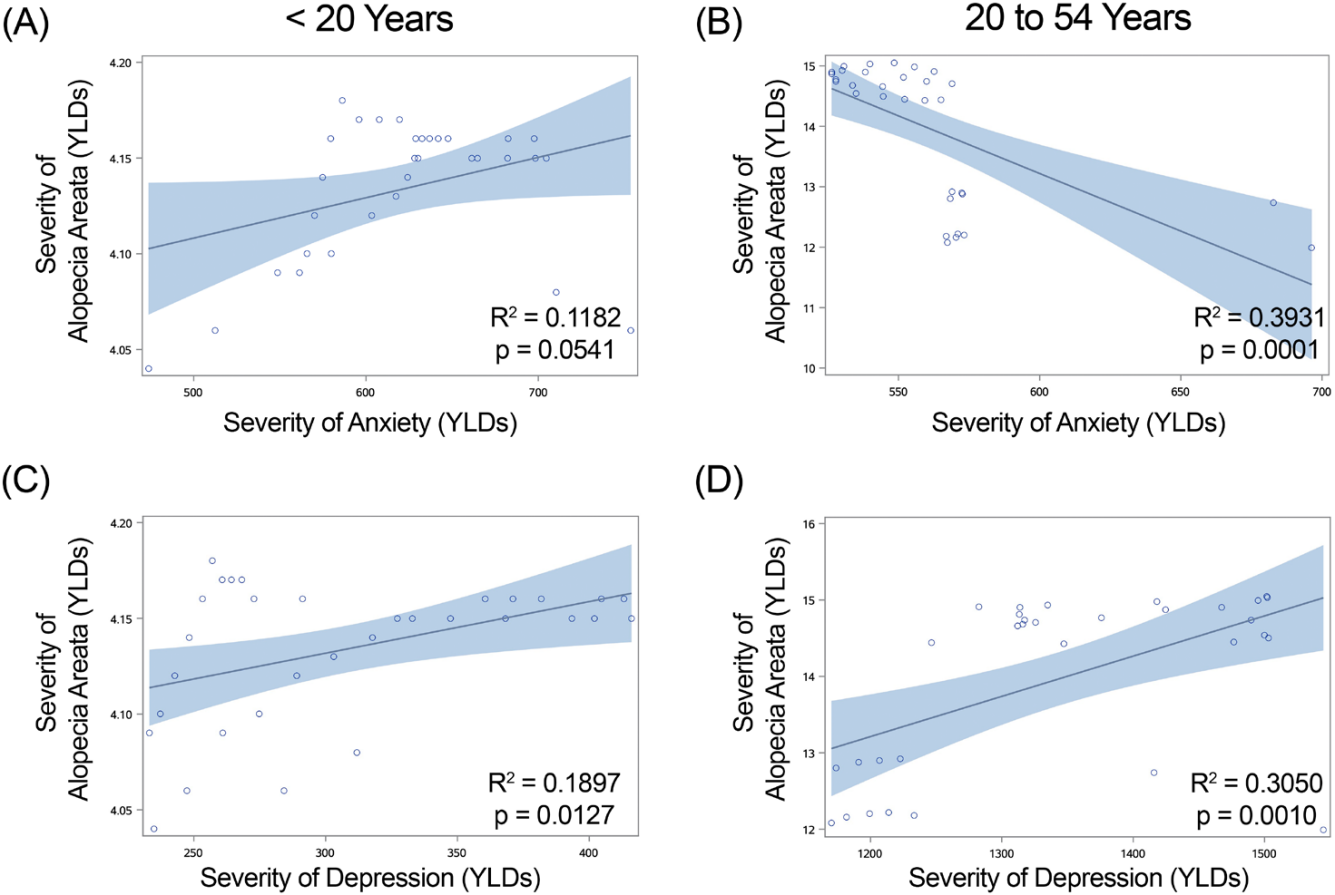
Regression of severity (Years Lived with Disability [YLDs]) of alopecia areata versus anxiety and depression in females within Brazil. Anxiety is shown in graphs (A, B) and depression in graphs (C, D). (A, C) females under 20 years of age; (B, D) females 20–54 years of age. *R* ^2^ and *p*‐values for each regression shown within each graph.

**FIGURE 6:**
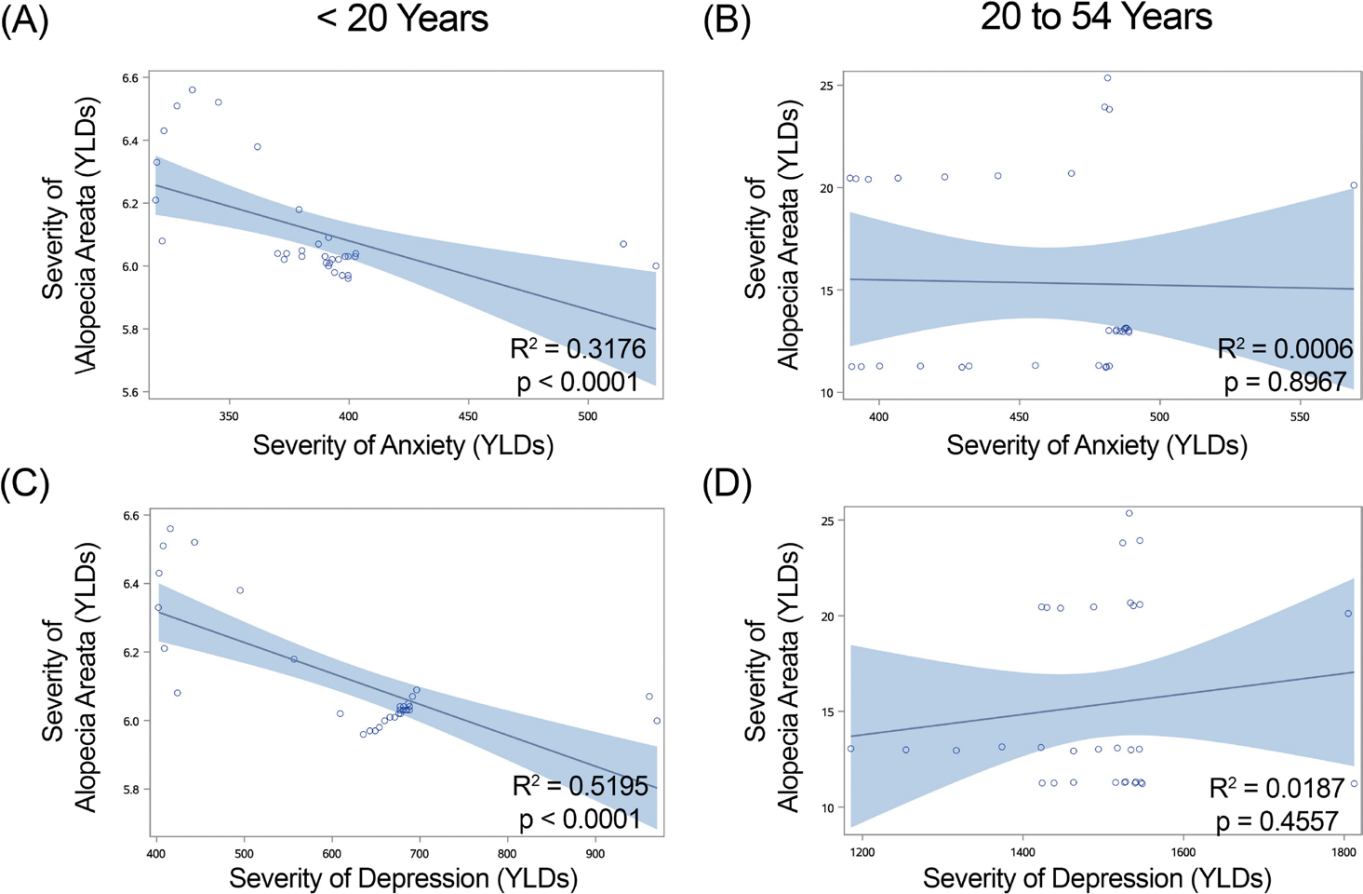
Regression of severity (Years Lived with Disability [YLDs]) of alopecia areata versus anxiety and depression in females within the United States. Anxiety is shown in graphs (A, B) and depression in graphs (C, D). (A, C) females under 20 years of age; (B, D) females 20–54 years of age. *R* ^2^ and *p*‐values for each regression shown within each graph.

We have examined the prevalence and the YLDs (years lived with disability) in our populations of interest. The prevalence is a measure of the extent of the disorder in the population of interest, including all cases whether existing or new in a particular year. The YLDs measure can be thought of as a surrogate for the severity of the disorder. In Figure 1 we present heat maps which highlight the range of high and low values of the Pearson's correlation coefficient graphically. These maps summarize the correlations and associated significance levels that we observed in our entire dataset, with values shaded towards red representing strong positive correlations, while values shaded towards blue represent strong negative correlations. In Figures 2, 3, 4, 5, 6, we show the data for YLDs in females under the age of 20 years and between the ages of 20 and 54 years in China, Japan, India, Brazil, and the United States, respectively. For brevity, we have chosen to only show graphs for these populations as the majority of the significant relationships are within these age groups.

### 3.1 Relation of Alopecia Areata With Anxiety Disorders

We found a significant positive correlation in females less than 20 years of age in China (Figure 2A), Japan (Figure 3A) and India (Figure 4A) for YLDs. In females between the ages of 20 and 54 years, we found a positive correlation for YLDs in China (Figure 2B, for YLDs). In females age 55 years and older, we found a significant positive correlation for prevalence in China (Figure 1A).

### 3.2 Relation of Alopecia Areata With Depressive Disorders

When we examined females under the age of 20 years, we found a significant positive correlation for YLDs in China (Figure 2C), India (Figure 4C) and Brazil (Figure 5C). In females aged 20–54 years, we observed significant positive correlations for YLDs in China (Figure 2D), India (Figure 4D) and Brazil (Figure 5D).

## 4 Discussion

We have demonstrated that there is a significant but complex relationship between alopecia areata and anxiety and depressive disorders. The novelty of this particular study is that we have analyzed the data broken down into 3 different age groups in males and females separately, allowing us to gain a better understanding of these complex relationships. Additionally, we have analyzed a subset of countries versus general regions, enabling us to compare socio‐cultural differences.

When we examine females under the age of 20 years, we find that there are significant positive associations in alopecia areata and anxiety disorder in females in China, Japan, and India for both prevalence and YLDs, and in Brazil for prevalence. However, we do not see this in males of the same age group. We continue to see a significant positive association between alopecia areata and anxiety in female individuals in China aged 20–54 years for both prevalence and YLDs, and in females 55 years of age and older for prevalence, but no significant positive associations are seen in males aged 20 to 54 years and 55 years of age and older.

When we investigated depression, we found significant positive correlations with alopecia areata in China, India, and Brazil in females under 55 years of age, but we did not observe the same relationships in males of any age group or country.

These results differ from what has been shown by Jang, et al. who demonstrated increased anxiety in both males and females, but a decrease in depressive disorders in males and females [10]. However, Jang et al. used age‐standardized rates and percentages, not allowing for the effect of age to be examined directly [10]. Additionally, Jang et al. only examined larger regions/continents, whereas we have examined individual countries. One potential reason for the differences we have observed between males and females may be explained by how hair loss is viewed between the sexes. Hair loss is generally seen as more socially acceptable in males than females [9, 12], but hair also forms part of the personal identity, particularly in females [8, 9]. Jang et al. and Jun et al. have both shown that emotional distress is in fact common in patients with alopecia areata [10, 13]; however, there may be differences in how men and women are able to cope with the stress of this appearance altering disorder.

The COVID‐19 pandemic helped highlight the difference in coping mechanisms between men and women, where women are more likely to experience anxiety [14]. A meta‐analysis of the trauma response differences between men and women showed increased vulnerability in women [15]. Additionally, in patients with androgenetic alopecia, men are able to cope more effectively than women, likely due to differences in self‐image [16]. Work by Hwang et al. has demonstrated quantitatively the impact of hair loss in females, showing higher Hair‐Specific Skindex‐29 scores in females compared to males [12].

Also, of interest is that in females in the United States (Figures 1 and 6), we do not see the same positive associations that we observe in China, Japan, Brazil, and India. This could be due to several factors. Firstly, there may be socio‐cultural differences in how hair loss is viewed in the countries we examined. In western countries, it has become more socially acceptable to have hair loss, particularly in men [17]. However, detailed research on the levels of stigmatization of hair loss between different regions in each of the sexes is scant. Even with scant research, there is evidence of stigmatization of people with alopecia, as demonstrated by Creadore et al. [7] Further research in this area may help determine these specific differences, with the goal of developing methods for reducing stigma and increasing patient well‐being. Another factor that may be influencing the differences we have observed is access to healthcare. This would include access to effective treatments, including but not limited to prescription medications, light therapies, and camouflaging techniques (e.g., wigs and hair pieces). Access to dermatological care is not equal across the globe [18, 19]. Factors affecting these disparities in access to care includeavailability of practitioners, public health policy (government vs. private systems), community context (e.g., general believes and attitudes of community), as well as others [20].

Another intriguing observation is that the correlations between alopecia areata and mental health disorders are more apparent in younger female populations, evidenced in Figure 1, where stronger positive correlations are seen more frequently in the less than the 20 years of age group. This skewing of correlation to younger ages may have several causes. Older patients, who have potentially lived with the disorder for a longer period of time may have adapted psychologically (or accepted) their condition, leading to lower levels of distress. Alternatively, these skewed correlations could also be due to patients in these older age groups having more pressing health concerns where alopecia areata, and even hair loss in general may not be a high priority in their lives.

It is also important to note that the relationship between autoimmune disorders such as AA, stress, and mental health is complex. Psychological stress, which can be triggered by the presence of hair loss, can not only lead to anxiety and depression, but can also directly impact the immune system and the level of inflammation in the body by disrupting the hypothalamus‐pituitary–adrenal axis, which regulates the stress response [21]. Additionally, there are sex differences in how the stress response impacts inflammation within the body [22], which is consistent with the results that we have presented. This provides a potential mechanism for the psychological stress that may be triggered by AA to further contribute to its exacerbation.

Our work has provided valuable insight into the psycho‐social impact of alopecia areata; however, there are limitations to the work that we have presented. Firstly, the data we have accessed in the global burden of disease study is aggregate data where we only have access to the rates of prevalence and YLDs for each country and age group for each year. Because of this data structure, we can only determine correlations between disorders, versus causal relationships. In addition, the measures that we have used are only proxies for the true extent and severity of these disorders, but nonetheless, these measures do provide meaningful information.

We also have not included environmental and behavioral factors in our study, which could have the potential to impact the results that were observed. Additionally, even though we have broken our analysis into three age groups for each sex, there are still refinements that could be done to further tease out the complexity of the relationship between these disorders. Along those same lines, we have only examined five countries, which limits the applicability of these results to the rest of the world. Future work could include more countries covering all continents/regions and political/social/cultural norms to gain more generalizability, but with the caveat that there is the potential for different results.

## 5 Conclusions

We have demonstrated that there is a complex relationship between alopecia areata and anxiety and depressive disorders, with correlations varying by country. There is an interplay between mental health and alopecia areata, where some patients may experience distress due to hair loss, while others may have pre‐existing anxiety or depression which could potentially be worsened by hair loss.

The impact of hair loss disorders such as alopecia areata on the mental health of patients should not be discounted, but rather addressed as part of a patient's ongoing care.

## Author Contributions

A.K.G. and V.E. wrote and critically reviewed the manuscript. V.E. extracted and analyzed the data. M.T. critically reviewed the manuscript.

## Ethics Statement

Specific ethics approval was not required for this study as no human subjects were directly involved in this research. All data that was accessed is freely available to the public at https://vizhub.healthdata.org/gbd‐results/.

## Conflicts of Interest

The authors declare no conflicts of interest.

## Data Availability

Data can be made available upon reasonable request.
